# OpenMS – An open-source software framework for mass spectrometry

**DOI:** 10.1186/1471-2105-9-163

**Published:** 2008-03-26

**Authors:** Marc Sturm, Andreas Bertsch, Clemens Gröpl, Andreas Hildebrandt, Rene Hussong, Eva Lange, Nico Pfeifer, Ole Schulz-Trieglaff, Alexandra Zerck, Knut Reinert, Oliver Kohlbacher

**Affiliations:** 1 Center for Bioinformatics, Eberhard Karls University Tübingen, Sand 14, 72076 Tübingen, Germany; 2 Algorithmic Bioinformatics, Free University Berlin, Takustr. 9, 14195 Berlin, Germany; 3 Max Planck Institute for Molecular Genetics, Ihnestr. 63-73, 14195 Berlin, Germany; 4 Center for Bioinformatics, Saarland University, Stuhlsatzenhausweg, Bld. E11, 66041 Saarbrücken, Germany

## Abstract

**Background:**

Mass spectrometry is an essential analytical technique for high-throughput analysis in proteomics and metabolomics. The development of new separation techniques, precise mass analyzers and experimental protocols is a very active field of research. This leads to more complex experimental setups yielding ever increasing amounts of data. Consequently, analysis of the data is currently often the bottleneck for experimental studies. Although software tools for many data analysis tasks are available today, they are often hard to combine with each other or not flexible enough to allow for rapid prototyping of a new analysis workflow.

**Results:**

We present OpenMS, a software framework for rapid application development in mass spectrometry. OpenMS has been designed to be portable, easy-to-use and robust while offering a rich functionality ranging from basic data structures to sophisticated algorithms for data analysis. This has already been demonstrated in several studies.

**Conclusion:**

OpenMS is available under the Lesser GNU Public License (LGPL) from the project website at .

## Background

Mass spectrometry is a key analytical technique for biomedical research in proteomics and metabolomics. The usual procedure is first to reduce the complexity of the sample by separation techniques, and then to conduct one or more mass spectrometric analyses. Common separation techniques are high performance liquid chromatography (HPLC), capillary electrophoresis (CE) or two-dimensional gel electrophoresis (e.g. 2D PAGE). Techniques like HPLC or CE have certain advantages over 2D PAGE for high throughput analysis, since they can be easily automated.

Variations of this setup are widely used in differential analysis of samples of healthy and diseased patients, to investigate changes occurring in time series experiments, or to explore the effects of perturbations to biological systems. This type of analysis is an essential tool for understanding the molecular foundations of diseases, the discovery of biomarkers or the identification of potential drug targets.

The keen interest of researchers in the field and the rapid improvement of analytical instruments opens the door to a variety of new and complex experimental setups producing large amounts of data. In recent years it has become evident that the handling and computational analysis of the data is the major bottleneck for biomedical studies in the field of proteomics or metabolomics.

Two of the main applications of mass spectrometry today are *identification *and *quantitation *of compounds. Although there exists a variety of software tools for those tasks, they are often monolithic and difficult to adapt to rapidly evolving demands. It is advantageous to view the problems as a series of smaller problems. For example, one specific quantitation protocol could consist of a process involving *peak picking, quantitation, normalization, dewarping *and *marker finding*. Other quantitation protocols use labeled data and hence differ from the above, although they have many steps in common. Therefore, it is desirable to have smaller algorithmic components that can be readily combined into more complex workflows or tools. OpenMS aims at providing exactly this functionality and is therefore more flexible than almost all current commercial tools and most of the openly available libraries.

Commercial software packages, e.g. MassLynx [1] and DeCyder [2], often perform specific tasks well, however, almost all rely on proprietary data formats and import/export of open data formats is not always possible. Moreover, the algorithms used are often not known to the public and the parameters that govern their behavior are rarely accessible. In summary, one can say that the use of commercial tools limits the control the user has over the data analysis.

There are plenty of academic projects for proteomics data analysis which use open data formats and give the user much more control over the steps of the analysis, e.g. MapQuant [3], MASPECTRAS [4], msInspect [5], MzMine [6], SpecArray [7], The OpenMS Proteomics Pipeline (TOPP) [8], Trans-Proteomic Pipeline (TPP) [9], Viper [10], Superhirn [11] and XCMS [12]. Nearly all these tools can read and write at least one of the two open standard data formats: mzData [13] and mzXML [14] proposed by the Human Proteome Organisation – Proteomics Standards Initiative (HUPO-PSI) and the Institute for Systems Biology (ISB), respectively. Most of the tools concentrate on one step of the analysis, e.g. quantitation, peptide identification or map alignment, or combine a few steps into a pipeline. The algorithms are usually published and the tools or the source-code are freely available for academic use.

Although many commercial and academic software tools are available, the existing tools are often not flexible enough to be adapted to new experiments because they have been developed for a specific use-case. Moreover, there are no efficient implementations of some known algorithms, because researchers refrain from turning a proof-of-concept implementation into a robust software tool for the community.

OpenMS was designed for rapid application development in proteomics data analysis. It serves as a framework for developing mass spectrometry data analysis tools, providing everything from basic data structures over file input/output (I/O) and visualization to sophisticated algorithms. Thus, OpenMS allows developers to focus on new algorithmic approaches instead of implementing infrastructure. In the next sections, we will highlight the core architectural features of OpenMS and how it can be used to construct powerful applications for proteomics data analysis. We will also elaborate the different algorithms already contained in OpenMS and demonstrate its versatility in several small code examples.

## Implementation

OpenMS is intended to offer a rich functionality while keeping in mind the design goals of ease-of-use, robustness, extensibility and portability. We will now briefly describe the techniques used to achieve these goals.

Furthermore, selected features of OpenMS are presented, especially those interesting for application developers. Small code pieces are used to give a first impression of OpenMS code.

### Design goals

#### Ease-of-use

The object-oriented programming paradigm aims at mapping real-world entities to comprehensible data structures and interfaces. Combining it with a coding style that enforces consistent names of classes, methods and member variables, leads to intuitive usability of a software library. For those reasons we adapted this paradigm for OpenMS. A second important feature of a software framework is documentation. We decided to use doxygen [15] to generate the class documentation from the source code, which ensures consistency of code and documentation. The documentation is generated in HTML format making it easy to read with a web browser. OpenMS also provides a tutorial that introduces the most important concepts and classes using example applications.

#### Robustness

While robustness is not of the essence when developing new algorithms, it is essential if a new method will be applied routinely to large scale datasets. Typically, there is a trade-off between performance and robustness. OpenMS tries to address both issues equally. In general, we try to tolerate recoverable errors, e.g. files that do not entirely fulfill the format specifications. On the other hand, exceptions are used to handle fatal errors. To check for correctness, unit tests are implemented for each method of a class. These tests check the behavior for both valid and invalid use. Additionally, preprocessor macros are used to enable additional consistency checks in debug mode, which are then disabled in productive mode for performance reasons.

#### Extensibility

Since OpenMS is based on several external libraries it is designed for the integration of external code. All classes are encapsulated in the *OpenMS *namespace to avoid symbol clashes with other libraries. Through the use of template code, many data structures are adaptable to specific problems. For example, it is possible to replace the representation of the mass-spectrometric peak or to replace the container a spectrum uses to store the peaks in. Also, OpenMS supports standard formats and is itself open-source software. The use of standard formats ensures that applications developed with OpenMS can be easily integrated into existing analysis pipelines. OpenMS source code is located on SourceForge [16], a repository for open-source software. This allows users to participate in the project and to contribute to the code base.

#### Portability

Currently, OpenMS can be compiled on most Unix-like platforms (e.g. MacOS, Solaris, Linux) and has been thoroughly tested on several Linux distributions. Through the use of ANSI C++, porting it to other platforms poses no major problem.

The second emphasis of OpenMS, besides the design goals, is rich functionality. The framework offers data structures to handle MS data and metadata. It supports visualization of the data, file I/O and database I/O. This more basic functionality is complemented by a variety of algorithms for data analysis. All key analysis steps like signal processing, quantitation and peptide identification are addressed. The overall architecture and some selected features are illustrated in the following sections.

### Overall architecture and features

**Figure 1:**
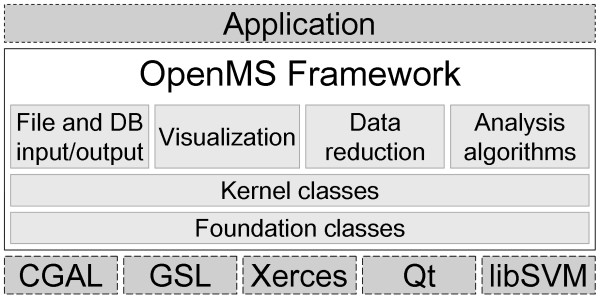
The overall design of OpenMS.

The overall design of OpenMS is shown in Fig. 1. Without looking into the details of OpenMS the situation is very simple. Applications can be implemented using OpenMS, which in turn relies on several external libraries: Qt [17] provides visualization, database support and a platform abstraction layer. Xerces [18] allows XML file parsing. libSVM [19] is used for machine learning tasks. The Computational Geometry Algorithms Library (CGAL) [20] provides data structures and algorithms for geometric computation. The GNU Scientific Library (GSL) [21] is used for different mathematical and statistical tasks.

OpenMS can itself be subdivided into several layers. At the very bottom are the foundation classes which implement low-level concepts and data structures. They include basic concepts (e.g. factory pattern, exception handling), basic data structures (e.g. string, points, ranges) and system-specific classes (e.g. file system, time). The kernel classes, which capture the actual MS data and metadata, are built upon the foundation classes. Finally, there is a layer of higher-level functionality that relies on the kernel classes. This layer contains database I/O, file I/O supporting several file formats, data reduction functionality and all other analysis algorithms.

**Figure 2:**
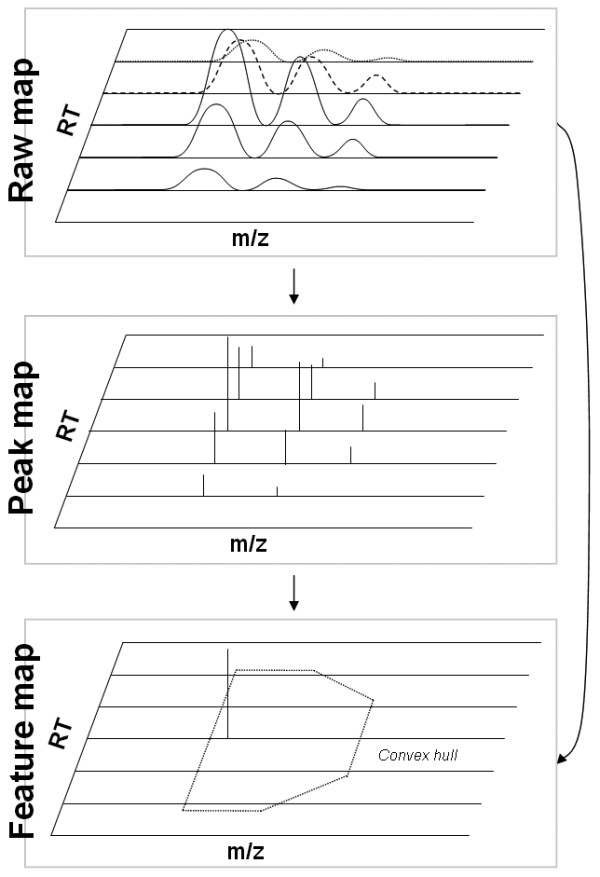
**The data reduction concept of OpenMS**. A raw map (top) which is first reduced to a peak map (middle) and finally to a feature map (bottom). The direct reduction of raw to feature map is also possible.

Data reduction is a central concept of OpenMS and will now be considered in more detail. Data reduction comprises two transformation steps as displayed in Fig. 2: The conversion of raw spectra (also called continuous spectra or profile spectra) to peak spectra (also called stick spectra) and the conversion of a raw or peak map (a collection of MS spectra produced by an experiment) to a feature map. By *feature *we denote the two-dimensional signal created by some chemical entity, e.g. a peptide or metabolite. A feature is characterized by its isotopic pattern in mass-to-charge dimension and by the elution profile in retention time dimension.

In the following sections, selected features of OpenMS are presented using small code examples.

### Kernel datastructures

The kernel datastructures (e.g. peaks, spectra, maps, features) are the base of each OpenMS application, which is why special care has been taken in their design. As stated before, these classes are customizable through the use of template code. For example, the peak type of spectra/maps can be changed according to the amount of information needed for a specific use case. This allows very efficient memory use – a 115 MB mzData file containing approximately 2.1 million raw data points can be stored in 27 MB of RAM on a 32 bit computer. The examples in the following sections will also illustrate usage of these kernel classes.

### Standardized file formats

Standardized data exchange formats are especially important because they allow the easy integration of different software tools into a single analysis pipeline. Therefore, OpenMS supports most non-proprietary file formats, e.g. mzData and mzXML. As there are no standard file formats for quantitation and peptide identification data, we created our own formats for these tasks (featureXML and idXML). Import and export of pepXML/protXML [9] is planned for the future. Eventually, our formats will be replaced by standard formats released by the HUPO-PSI. Currently, we are actively contributing to the development of the upcoming standards mzML and analysisXML. mzML is intended to replace both the mzData and the mzXML format. analysisXML captures the results of peptide and protein search engines.

The following listing demonstrates how easily one can convert between mzData and mzXML format using the respective OpenMS classes:

1 PeakMap map;

2 MzDataFile infile;

3 infile.load("example.mzData", map);

4 MzXMLFile outfile;

5 outfile.store("example.mzXML", map);

In line 1 the data structure that holds the data is created. In lines 2 and 3 the input file adapter is instantiated and used to fill that map variable with the data from a mzData file. In lines 4 and 5 the data is written to an mzXML file using the corresponding file adapter.

### Database support

Currently, most tools developed operate on files. Because of the constantly growing data volume created by HPLC-MS experiments, database systems will become more and more important for data management. Therefore, we developed a database adapter that can persistently store the kernel data structures in an SQL database. Through the use of Qt database adapters as an additional layer of abstraction, the implementation is able to employ most SQL compliant relational database management systems (including MySQL, PostgreSQL, ORACLE and DB2). The interface of the database adapter is very similar to the file adapters, which is why we omit an example.

### Visualization

**Figure 3:**
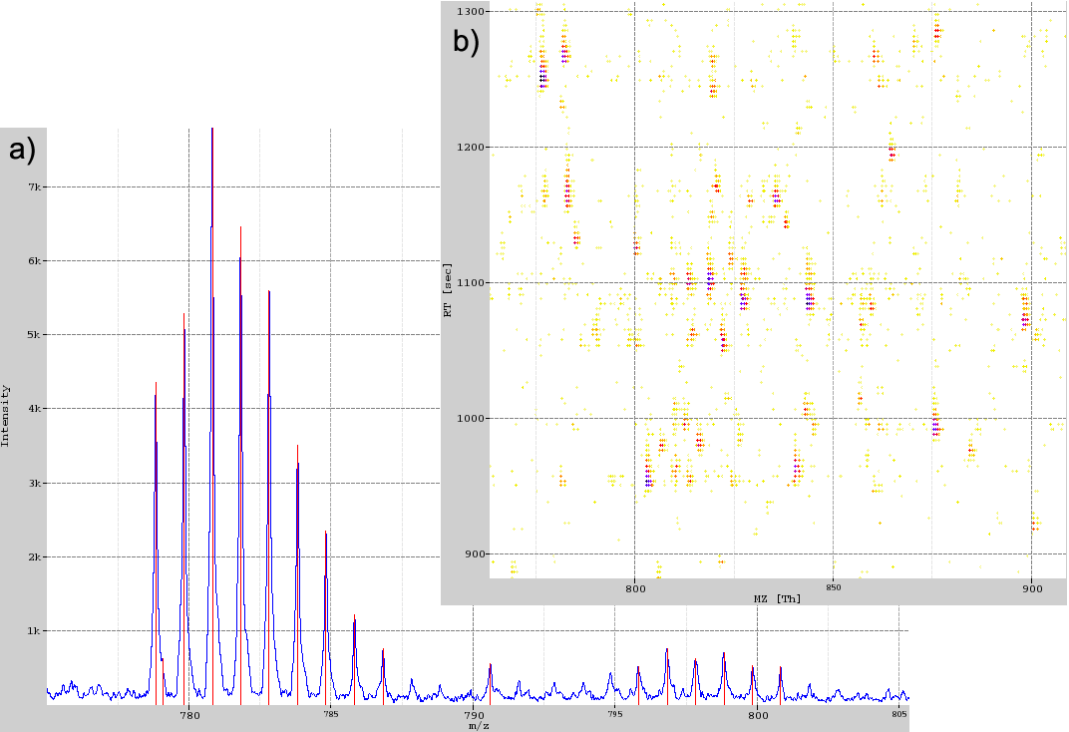
**Visualization widget examples**. (a) Visualization of a raw data spectrum and the corresponding peaks spectrum as two superimposed layers. (b) Part of an HPLC-MS map displayed in a 2D view.

A very useful tool for data analysis is visual inspection. It can instantly reveal properties of the data that would go unnoticed using command line tools. Errors during separation or polymeric contamination of the sample can, for example, be easily noticed during visual inspection of an HPLC-MS map. OpenMS provides widgets that display a single spectrum or a peak map. A single spectrum is displayed by a standard plot of raw or peak data. A peak map is displayed either in a 2D view from a bird's-eye perspective with color-coded intensities or in a 3D view. Fig. 3 shows an example of the spectrum and the 2D map view.

In order to display two given spectra as shown in Fig. 3(a) the following code could be used:

1 PeakMap map1, map2;

2 ...// Fill the maps with data

3 Spectrum1DWidget* gui;

4 gui = new Spectrum1DWidget(Param());

5 gui->canvas()->addLayer(map1);

6 gui->canvas()->addLayer(map2);

In line 1 two maps are created which should be filled with data in a real-world application but remain empty in this toy example. In lines 3 and 4 the widget used for visualization is created with an empty set of parameters. Therefore, default parameters (e.g. peak color, background color) are used. Finally, both maps are added to the canvas sub-widget. Handing over a map to the widget, although it displays only one spectrum, is necessary, because all spectrum and map visualization widgets share a common base class and thus a common interface.

### Signal processing

OpenMS offers several filters to reduce chemical and random noise as well as baseline trends in MS measurements. Raw spectra may either be de-noised by a Savitzky-Golay filter or a peak-area-preserving Gaussian low-pass filter. Both smoothing filters are commonly used and recommended for spectrometric data [22,23]. For the baseline in MS experiments, no universally accepted analytical expression exists. Hence, we decided to implement a nonlinear filter, known in morphology as the top-hat operator [24]. This filter does not depend on the underlying baseline shape and its applicability to MS measurements has already been shown in [25]. For extraction of the accurate information about the mass spectral peaks in a raw spectrum we developed an efficient peak picking algorithm [26] that uses the multi-scale nature of spectrometric data. First, the peak positions are determined in the wavelet-transformed signal. Afterward, important peak parameters (centroid, area, height, full-width-at-half-maximum, signal-to-noise ratio, asymmetric peak shape) are extracted by fitting an asymmetric peak function to the raw data. In an optional third step, the resulting fit can be further improved by using techniques from nonlinear optimization. In contrast to currently established techniques, our algorithm yields accurate peak positions even for noisy data with low resolution and is able to separate overlapping peaks of multiply charged peptides.

The next code example demonstrates a small analysis pipeline consisting of a baseline reduction and a peak picking step:

1 RawMap exp_raw;

2 ...// Fill the map with data

**Figure 4:**
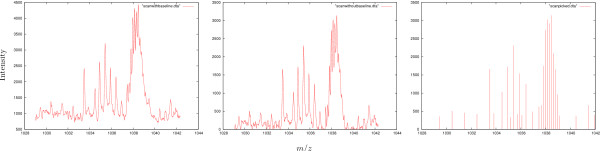
**Signal processing example**. Result of baseline reduction and peak picking: From the raw spectrum (left) the baseline is subtracted (middle) and then peaks are picked (right).

3 RawMap exp_filtered;

4 TopHatFilter th;

5 th.filterExperiment(exp_raw, exp_filtered);

6 PeakMap exp_picked;

7 PeakPickerCWT pp;

8 pp.pickExperiment(exp_filtered, exp_picked);

After filling the PeakMap the raw data are filtered using the TopHatFilter. The filtered data are stored in exp_filtered. Then the peaks are picked and stored in exp_picked. In this example no parameters are explicitly set, so the default parameters are used. Fig. 4 visualizes the effect of the above program on a single raw data spectrum.

### Feature detection and quantitation

Feature detection is a central concept in OpenMS. As noted before, a feature is a signal in an HPLC-MS map which is e.g. caused by a peptide ion. Each feature is characterized by its mass-to-charge ratio, the centroid of its elution curve and the signal area.

**Figure 5:**
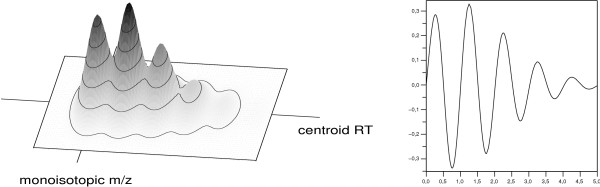
**The OpenMS feature model**. The averagine model (left) and the corresponding isotope wavelet (right) used by our algorithm. (Shown for mass ~2000 Da and charge 1.).

OpenMS includes several algorithms for the detection of peptidic features in HPLC-MS data, tailored for datasets of different mass resolutions and measured on various instrument types. Our approaches are based on a two-dimensional model. We use the concept of an average amino acid (also called *averagine*) to approximate the amino acid composition for a peptide of a given mass. From this we can estimate its atomic composition and derive its isotope distribution in a mass spectrum [27]. Similarly, we approximate the elution curve by a Gaussian or exponentially modified Gaussian distribution [28]. The combined two-dimensional model can be seen in Fig. 5 (left). In addition, our isotope pattern model takes different mass resolutions into account by incorporating a parameter for the width of the isotopic peaks in a feature.

Fitting the two-dimensional model is a relatively expensive computational task. Therefore, it is important to select the candidate regions carefully. We designed a novel algorithm [29] that uses a hand-tailored isotope wavelet [30] to filter the mass spectra for isotopic patterns for a given charge state. The isotope wavelet explicitly models the isotope distribution of a peptide; see Fig. 5 (right). This pre-filtering results in a lower number of potential peptide candidates that need to be refined using the model fit.

The next code example demonstrates how the feature detection can be used.

1 RawMap exp_raw;

2 ...// Fill the map with data

3 FeatureMap<> features;

4 FeatureFinder ff;

5 ff.run("simple", exp_raw, features, Param());

The experimental raw data are stored in a RawMap, and the extracted features in a FeatureMap. To quantify, we invoke the run method of the FeatureFinder. It is a template function therefore other data structures for input and output are supported as well. Various types of algorithms are available – in this case we choose "simple". As in the examples before, algorithm parameters are handed over in a Param object. Each algorithm has a different set of parameters, which can be read from a file in XML format or set in the program.

### Data alignment

An important step in a typical HPLC/MS or CE/MS analysis workflow is the combination of results from multiple experiments, e.g. to improve confidence in the obtained measurements or to compare results from different samples. In order to do so, a suitable mapping or *alignment *between the datasets needs to be established. The alignment has to correct for (random and systematic) variations in the observed elution time and mass-to-charge ratio that are inevitable in experimental datasets.

**Figure 6:**
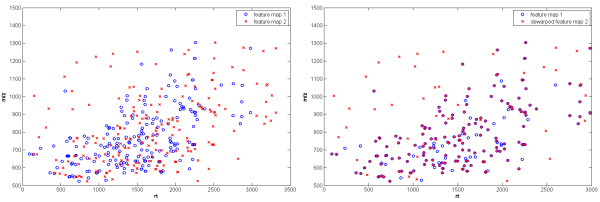
**Map alignment example**. Left: Two feature maps with varying retention time and mass-to-charge dimensions. Right: The features of the second feature maps were transformed onto the coordinate system of the first feature map.

OpenMS offers algorithms to align multiple experiments and to match the corresponding ion species across many samples. A novel and generic algorithm was developed to correct for the variation of retention time and mass-to-charge dimensions between two maps. It uses an adapted pose clustering approach [31,32] to efficiently superimpose raw maps as well as feature maps. In Fig. 6 two feature maps are shown. In the left plot the retention times and the mass-to-charge ratio of corresponding features vary extremely and corresponding ion species are hard to determine. However, after the mapping of the two feature maps onto a consistent coordinate system the correspondence between the two maps can easily be seen in the right plot.

To detect and combine corresponding features in multiple feature maps into a so-called *consensus map*, we developed an algorithm based on techniques from computational geometry. The superimposition algorithm and the algorithm for the determination of a consensus map are combined to a star-wise approach for the alignment of multiple raw or feature maps. The overall methods are fast, reliable, and robust, even in the presence of many noise signals and large random fluctuations of retention time. Details of the map alignment algorithms can be found in [33].

In the following code example three feature maps are aligned and the resulting consensus map is stored in a file.

1 FeatureMap fm1, fm2, fm3;

2 ...// Fill the feature maps with data

3 StarAlignment<ConsensusFeature> alignment;

4 alignment.getElementMapVector().push_back(&fm1);

5 alignment.getElementMapVector().push_back(&fm2);

6 alignment.getElementMapVector().push_back(&fm3);

7 alignment.run();

8 ConsensusXMLFile file;

9 file.store("ouptut.xml", alignment);

After instantiating three feature maps in line 1 they are filled with data in line 2. In line 3 a StarAlignment object that is specialized for the alignment of feature maps is instantiated. The three feature maps are assigned to the alignment object in lines 4 to 6 and the actual alignment procedure is invoked in line 7. To store the resulting consensus map in ConsensusXML format we use the appropriate file handler in lines 8 and 9.

### Retention time prediction

A major problem with existing tandem mass spectrometry identification routines lies in the significant number of false positive and false negative annotations. Until now, standard algorithms for protein identification have not used the information gained from separation processes usually involved in peptide analysis, such as retention time information, which are readily available from chromatographic separation of the sample. Identification can thus be improved by comparing measured retention times to predicted retention times. Current prediction models are derived from a set of measured test analytes but they usually require large amounts of training data.

OpenMS offers a new kernel function, the *paired oligo-border kernel (POBK)*, which can be applied in combination with support vector machines to a wide range of computational proteomics problems. This enables the user to predict peptide adsorption/elution behavior in strong anion-exchange solid-phase extraction (SAX-SPE) and ion-pair reversed-phase high-performance liquid chromatography (IP-RP-HPLC). Using the retention time predictions for filtering significantly improves the fraction of correctly identified peptide mass spectra. OpenMS offers a wrapper class to the libsvm [19] for support vector learning. Our *POBK *is well-suited for the prediction of chromatographic separation in computational proteomics and requires only a limited amount of training data. Usually 40 peptides or less are sufficient. A more detailed description of the methods for retention time prediction, as well as the application of the retention time prediction to improve tandem MS identification results, can be found in [34]. The following code example shows how retention times can be predicted:

1 SVMWrapper svm;

2 vector<DoubleReal> predicted_rts;

3 svm_problem* training_data = NULL;

4 svm_problem* test_data = NULL;

5 ...// Load and encode data

6 ...// Set parameters of svm

7 svm.train(training_data);

8 svm.predict(test_data, predicted_rts);

After loading and encoding the data using the LibSVMEncoder class, one has to set the parameters of the support vector machine. It is also possible to determine the best parameters by performing a cross validation over parameter ranges. Afterwards the svm can be trained. The trained svm can then be used to predict retention times. At the end of the code example the predicted retention times are stored in the predicted_rts vector.

## Results

In this section we provide an idea of the kind of analyses that can be conducted with applications based on OpenMS in order to convey the versatility of our framework. Most of the studies are already published elsewhere and details can be found in the corresponding publications.

### TOPP – The OpenMS Proteomics Pipeline

OpenMS has been successfully used for the implementation of TOPP – The OpenMS Proteomics Pipeline [8]. TOPP is a set of computational tools that can be chained together to tailor problem-specific analysis pipelines for HPLC-MS data. It transforms most of the OpenMS functionality into small command line tools that are the building blocks for more complex analysis pipelines. The functionality of the tools ranges from data preprocessing (file format conversion, baseline reduction, noise reduction, peak picking, map alignment,...) over quantitation (isotope-labeled and label-free) to identification (wrapper tools for Mascot [35], Sequest [36], InsPecT [37] and OMSSA [38]).

The source code of the TOPP tools is on average not larger than 150 lines and the largest part of it deals with the evaluation of command line parameters. The core functionality of most of the tools can be implemented in less than 20 lines of code. This is possible not only because OpenMS facilitates file handling and offers clear interfaces for its algorithms. Moreover, it also offers powerful parameter handling classes both for parameters of algorithms and command line arguments of applications. For algorithms, there is a base class that provides a generic interface for string, float and integer parameters along with their documentation. It handles default parameters and can even be used to generate the parameter documentation automatically. For command line tools the TOPP base class, which implements the whole command line argument handling, is provided. The only thing the developer has to do is to specify the parameters along with their descriptions and default values. Given such an infrastructure, the development time of new tools is significantly reduced.

### Protein quantitation

OpenMS contains several algorithms for peptide quantitation based on model fitting [29,39]. Using the data structures provided by OpenMS and these algorithms, we were able to implement data analysis code for various complex quantitation tasks (labeled/unlabeled strategies, relative/absolute quantitation). In a case study [40] we could thus show that the use of these algorithms improved quantitation accuracy in a complex absolute quantitation scenario (myoglobin in human blood plasma) while drastically reducing analysis times.

### Protein and peptide identification

We illustrate the capability of OpenMS to identify peptides and proteins, given their tandem MS spectra, by the following example. Experimental data used in the analysis was a mixture of standard proteins contained in various concentrations (data not shown).

Kapp *et al*. [41] showed that most of the correct identifications are found by various search engines. Nevertheless, they also demonstrated that there is a certain amount of correctly identified spectra which could only be identified by one or two of the search engines which they compared in their study. For these reasons we integrated the identifications of different search engines like Mascot [35], InsPecT [37] and OMSSA [38]. The results of the different search engines were combined by calculating an average rank for the identification candidates. Tightening the used score thresholds of the different search engines results in more accurate identification, as only high quality identifications are kept. Using a looser score threshold the number of correctly identified peptides increases.

To find more true positive identifications one can lower score thresholds of the search engines further and filter out most of the additional false positive identifications using our retention time filtering approach. In this approach the retention time prediction model, already mentioned in subsection 'Retention time prediction', is trained by a small set of high confidence identifications. This model is then used to predict retention times for all further identifications. If there is a large difference between observed and predicted chromatographic behavior for some identifications, these identifications are excluded. This evaluation is described in more detail in [34].

All the steps mentioned are either implemented directly in OpenMS, like the consensus calculation, or are available as TOPP tools i.e. the search engine wrapper tools.

## Discussion

We have presented OpenMS – a large, versatile and functional software framework that is under active development as a joint project of several research groups. After more than three years of development in which we continuously improved and augmented the library, OpenMS 1.0 was released in July 2007 which improved tremendously upon the intermediate beta versions (e.g. see [42]). In its current state OpenMS can dramatically cut down on development time for devising analysis pipelines and testing new algorithmic strategies in the field of MS-based proteomics and metabolomics. Currently OpenMS offers about 350 classes that add up to more than 100.000 lines of code. We anticipate that OpenMS will contribute to speeding up biomedical research and bring the results of algorithmic research into an important area of science.

Developers using OpenMS are strongly encouraged to take part in the project by contributing their algorithms. Providing an algorithm in a framework would allow a much more flexible reuse than providing an application only.

Driven by collaborative projects with experimental partners we will add more functionality as the project proceeds. Projects for the near future comprise automatic algorithm parameter estimation for arbitrary experimental data, improved feature detection algorithms and porting OpenMS to Microsoft Windows.

## Availability and requirements

**Project name: **OpenMS

**Project home page: **

**Operating system(s): **Platform-independent (OpenMS can be compiled on most Unix-like platforms using an ANSI C++- compliant compiler)

**Programming language: **C++

**Software requirements: **Qt 4.1 or higher, OpenMS contrib library package

**Recommended hardware: **2 GB of disc space, 768 MB RAM

**License: **GNU Lesser General Public License (LGPL)

**Any restrictions to use by non-academics: **see LGPL license

**Documentation: **The class documentation is available in HTML format. The OpenMS and TOPP tutorials are available in HTML/PDF format.

## Authors' contributions

OK and KR initiated and coordinate the project. All authors contributed to the overall design and implementation of the library: MS implemented the kernel data structures, metadata handling, file I/O classes and visualization classes. Additionally, he maintains the build system and the unit test system. CG, KR and OST designed and implemented the feature detection algorithms. AH and RH contributed the isotope wavelet. EL and AZ designed and implemented the signal processing and peak picking algorithms. EL and CG designed and implemented the map alignment algorithms. AB, NP, OK and MS designed and implemented the algorithms related to peptide identification and retention time prediction. All authors contributed to, read, and approved the final version of this manuscript.
